# Clinical trial designs for rare diseases: Studies developed and discussed by the International Rare Cancers Initiative

**DOI:** 10.1016/j.ejca.2014.10.027

**Published:** 2015-02

**Authors:** Jan Bogaerts, Matthew R. Sydes, Nicola Keat, Andrea McConnell, Al Benson, Alan Ho, Arnaud Roth, Catherine Fortpied, Cathy Eng, Clare Peckitt, Corneel Coens, Curtis Pettaway, Dirk Arnold, Emma Hall, Ernie Marshall, Francesco Sclafani, Helen Hatcher, Helena Earl, Isabelle Ray-Coquard, James Paul, Jean-Yves Blay, Jeremy Whelan, Kathy Panageas, Keith Wheatley, Kevin Harrington, Lisa Licitra, Lucinda Billingham, Martee Hensley, Martin McCabe, Poulam M. Patel, Richard Carvajal, Richard Wilson, Rob Glynne-Jones, Rob McWilliams, Serge Leyvraz, Sheela Rao, Steve Nicholson, Virginia Filiaci, Anastassia Negrouk, Denis Lacombe, Elisabeth Dupont, Iris Pauporté, John J. Welch, Kate Law, Ted Trimble, Matthew Seymour

**Affiliations:** a European Organisation for Research and Treatment of Cancer, Belgium; b Medical Research Council Clinical Trial Unit at University College London, United Kingdom; c Cancer Research UK, United Kingdom; d Memorial Sloan Kettering Cancer Center, United States; e The University of Texas M.D. Anderson Cancer Center, United States; f The Royal Marsden, United Kingdom; g Hubertus Wald Tumorzentrum – University Cancer Centre Hamburg, Germany; h The Institute of Cancer Research, London, United Kingdom; i The University of Liverpool, United Kingdom; j The University of Cambridge Department of Oncology and NIHR Cambridge Biomedical Research Centre, United Kingdom; k Léon Bérard Cancer Center, France; l Cancer Research UK Clinical Trials Unit, Institute of Cancer Sciences, University of Glasgow, United Kingdom; m NIHR University College London Hospitals Biomedical Research Centre, United Kingdom; n The University of Birmingham, United Kingdom; o Istituto Nazionale dei Tumori Milan, Italy; p Cancer Research UK Clinical Trials Unit and MRC Midland Hub for Trials Methodology Research, University of Birmingham, United Kingdom; q The University of Manchester, United Kingdom; r Queen’s University Belfast, United Kingdom; s Mayo Clinic, United States; t Centre hospitalier universitaire vaudois, Switzerland; u Locum Consultant in Medical Oncology, United Kingdom; v Gynecologic Oncology Group/NRG Oncology, United States; w Center for Global Health, US National Cancer Institute, United States; x Institut National Du Cancer, France; y National Institute for Health Research Clinical Research Network/Cancer, United Kingdom; z Robert H. Lurie Comprehensive Cancer Center of Northwestern University, United States; aa Geneva University Hospital, Medical Oncology, Switzerland; ab The University of Nottingham, United Kingdom; ac Mount Vernon Cancer Centre, United Kingdom

**Keywords:** Rare cancers, Clinical trials, Randomised controlled trials, Methodology, Frequentist, Bayesian, Multi-arm

## Abstract

**Background:**

The past three decades have seen rapid improvements in the diagnosis and treatment of most cancers and the most important contributor has been research. Progress in rare cancers has been slower, not least because of the challenges of undertaking research.

**Settings:**

The International Rare Cancers Initiative (IRCI) is a partnership which aims to stimulate and facilitate the development of international clinical trials for patients with rare cancers. It is focused on interventional – usually randomised – clinical trials with the clear goal of improving outcomes for patients. The key challenges are organisational and methodological. A multi-disciplinary workshop to review the methods used in ICRI portfolio trials was held in Amsterdam in September 2013. Other as-yet unrealised methods were also discussed.

**Results:**

The IRCI trials are each presented to exemplify possible approaches to designing credible trials in rare cancers. Researchers may consider these for use in future trials and understand the choices made for each design.

**Interpretation:**

Trials can be designed using a wide array of possibilities. There is no ‘one size fits all’ solution. In order to make progress in the rare diseases, decisions to change practice will have to be based on less direct evidence from clinical trials than in more common diseases.

## 1 Introduction

The past three decades have seen rapid improvements in the diagnosis and treatment of cancer, and consequently in survival and other outcomes for cancer patients. Many factors have contributed to this progress, including public education and screening for earlier diagnosis, better access to diagnostic and treatment services, improved training and quality control in treatment delivery and improved supportive care.

The most important contributor to progress has been research, with public and private sector investment in preclinical and clinical research leading to rapid expansion of the evidence-base. For example, the introduction of a United Kingdom (UK) government-supported National Cancer Research Network from 2001 led to >5-fold increase in the number of cancer patients participating in research, so that ∼20% of all cancer patients participate in a national portfolio of studies.

Research activity has unsurprisingly focused on common cancers: industry prioritises cancers with the largest potential market and public sector funders prioritise those with the greatest clinical need. Furthermore, organising and delivering adequately-powered studies requires sufficient patients and a credible trial within a reasonable timescale can be infeasible in a rare cancer in a single country. Consequently, treatment is often based on insufficient evidence, and access to innovative drugs and technologies for research is poor.

This presents a major public health challenge. Rare cancers (incidence <6/100,000/year) [1] are a paradoxically common problem, accounting for 22% of all cancer diagnoses, higher than any single common cancer. But median survival for patients with rare cancer is typically poor and, unlike most common cancers, it is not improving.

The International Rare Cancers Initiative – IRCI – was formed in 2011 as a partnership between the National Institute of Health Research Cancer Research Network (NCRN) in England, Cancer Research UK, the Europe an Organisation for Research and Treatment of Cancer (EORTC) and the United States of America (USA) National Cancer Institute Cancer Therapy Evaluation Program (CTEP), and was joined in 2013 by the French National Institute of Cancer (INCa). IRCI’s aim is to stimulate and facilitate the development of international clinical trials for patients with rare cancers. It is focused on interventional – usually randomised – clinical trials with the clear goal of improving outcomes for patients.

IRCI faces two important challenges. The first is organisational: bringing together researchers from many countries, achieving consensus and overcoming the many regulatory and financial barriers which can impede the smooth running of international clinical research. The second is methodological: even with international collaboration, standard trial designs may require unfeasibly large recruitment targets for the setting, which calls for innovative methodologies to research.

A multi-disciplinary workshop to review the methods used in ICRI portfolio trials was held in Amsterdam in September 2013. Other as-yet unrealised methods were also discussed. Here, we present some of the methods available and illustrate them with examples from the IRCI portfolio. The aim is that researchers may consider these for use in future trials and understand the choices made for each design.

## 2 Methods, findings

The IRCI trials are each presented to exemplify one possible approach to the challenges of designing credible trials in rare cancers. Several trials used multiple such approaches, as will be clear from the description (Table 1, Table 2)^3^.

**Table 1:** Trial names and registration.

Short name	Full name	Registration number
BALLAD/IRCI 002	A study to evaluate the potential benefit of adjuvant chemotherapy for small bowel adenocarcinoma (SBA)	Pending application
Androgen deprivation therapy in advanced SGCs/IRCI 007	A randomised phase II study to evaluate the efficacy and safety of chemotherapy (CT) versus androgen deprivation therapy (ADT) in patients with recurrent and/or metastatic, androgen receptor (AR) expressing, salivary gland cancer (SGCs)	NCT01969578
HGUS/IRCI 006	A randomised double-blind phase II study evaluating the role of maintenance therapy with cabozantinib in High Grade Undifferentiated Uterine Sarcoma (HGUS) after stabilization or response to doxorubicin +/- ifosfamide following surgery or in metastatic first line treatment	EudraCT 2013-000762-11; NCT01979393
InterAACT/IRCI 003	An **Inter**national multicentre open label randomised phase II **A**dvanced **A**nal **C**ancer **T**rial comparing cisplatin plus 5-fluorouracil versus carboplatin plus weekly paclitaxel in patients with inoperable locally recurrent or metastatic disease	NCT02051868
rEECur	Trial of chemotherapy for relapsed and refractory Ewing sarcoma	ISRCTN36453794
GOG-0277/IRCI 001	A phase III randomised trial of gemcitabine plus docetaxel followed by doxorubicin versus observation for uterus-limited, high-grade uterine leiomyosarcoma	EudraCT 2012-002852-17; NCT01533207
MEKi ± AKTi in UM/IRCI 005	A randomised two-arm Phase II study of Trametinib alone and in combination with GSK2141795 in patients with advanced uveal melanoma	EudraCT number – 2013-002925-50; NCT01979523
InPACT/IRCI 004	International Penile Advanced Cancer Trial	NCT02305654

**Table 2:** Trial descriptions.

Short name	• Population	Incidence (per 100,000/year)	Concise description of the research question(s)	Sample size (patients)	Notes on trial design (Purpose, Primary end-point, Design type)
BALLAD/ IRCI 002	• Small bowel adenocarcinoma • Stage I-III • Fully resected • Fit for adjuvant fluoropyrimidine-based chemotherapy	2.2–5.7 (Western countries)	Q1-Adjuvant treatment versus conservative management where uncertainty of chemotherapy Q2-Fluoropyrimidine ‘monotherapy’ versus fluoropyrimidine + Oxaliplatin where chemotherapy will be given	Q1: 300 – 455 Q2: 280–425	Efficacy Disease Free Survival Uses a 20% 1-sided alpha and 80–90% power Hazard ratio refined using an external expert opinion prior (Bayesian)
Androgen deprivation therapy in advanced SGCs/ IRCI 007	• Salivary gland cancer • Histologically proven salivary duct cancer or adenocarcinoma NOS (not otherwise specified) • Recurrent and/or metastatic • Treatment naïve • Androgen receptor (AR) expression ⩾ 70% nuclei of neoplastic cells	2.5–3.0 (Western countries)	Androgen deprivation therapy (ADT) versus chemotherapy (CT)	76	Efficacy Progression Free Survival Korn design: inflated alpha at 10% 1-sided Target increase PFS rate at 6 months from 60% to 75%
HGUS/IRCI 006	• High-grade uterine sarcoma • Locally advanced (stage III-IV) or residual disease after primary surgery) or metastatic • Non-progressive at end of standard chemotherapy	0.07	Maintenance cabozantinib versus not	78	Activity Progression Free Survival Randomised blinded comparative phase II trial (Korn design, inflated alpha at 15% 1-sided) Target increase PFS at 4 months from 50% to 80%
InterAACT/IRCI 003	• Inoperable locally recurrent or • Metastatic squamous cell carcinoma of the anus (SCCA) • First line treatment	1.5–1.7 (CRUK and SEER data)	Cisplatin plus 5-FU versus carboplatin plus weekly paclitaxel	80	Set standard care; feasibility for future Response Rate Simon, Wittes & Ellenberg randomised selection trial design

rEECur	• Ewing sarcoma • Relapsed/refractory	0.09	3 chemotherapy doublets and 1 arm single agent (high dose)	∼500	Efficacy Interim: Response Rate; Final: Event Free Survival Multi-arm and multi-stage with arms dropping out by forced decisions Probability-based interpretation
GOG-0277/IRCI 001	• Uterine Leiomyosarcoma • Uterus-limited, High-grade	0.4–0.64	Adjuvant gemcitabine plus docetaxel followed by doxorubicin versus observation	216	Efficacy Overall Survival 80% power to detect HR = 0.625 at 5% 1-sided significance
MEKi +/- AKTi in UM/IRCI 005	• Uveal melanoma • Metastatic • No previous therapy for metastatic disease	0.43	Two experimental targeted therapies evaluated: either trametinib 208 plus GSK2141795 (MEK and AKT inhibition) or trametinib alone (MEK inhibition)	80	Activity Interim: Response Rate; Final: Progression Free Survival Randomization between 2 experimental arms 80% power to detect HR = 0.56 at 5% 1-sided significance
InPACT/ IRCI 004	• Squamous carcinoma of the penis who have inguinal lymph node metastases (i.e. locally-advanced disease) • No previous chemotherapy or chemoradiotherapy	1.5 (UK) 1570 cases in 2013 in US	1. role of neoadjuvant therapy and should it be chemotherapy or chemoradiotherapy 2. role of prophylactic pelvic lymph node dissection (PLND) following ILND in patients at high risk of recurrence?	400	Efficacy Overall Survival Bayesian design incorporating two sequential randomisations

### 2.1 Utilise a phase II design (Anal cancer)

One choice in the absence of sufficient patients for a phase III trial is to use a randomised phase II design to develop a collaboration for the future, as in InterAACT, an international, multicentre, open-label, randomised controlled trial (RCT). It is the first prospective trial of first-line treatment for patients with inoperable locally-recurrent or metastatic squamous cell carcinoma of the anus (SCCA). Eighty patients will be randomised to either cisplatin 5-FU or carboplatin + paclitaxel.

Age-standardised incidence rates in 2011 for new cases of SCCA were 1.5 (UK) and 1.7 (USA) per 100,000, comprising 0.4% of new cancers [2], [3]. Local relapse occurs in ∼20% patients treated with chemoradiotherapy; metastases in 10–17%. According to the Surveillance, Epidemiology and End Results (SEER) Program, estimated 5-year survival is 32% with metastatic disease [2].

The main aims of InterAACT are to: provide prospective randomised evidence for first-line treatment of inoperable locally advanced or metastatic SCCA; establish the optimal chemotherapy backbone for combination with new targeted agents for future trials; allow further exploration of tumour biology; promote the future development of selective therapeutic strategies; and establish set-up and recruitment feasibility of international SCCA trials. The longer-term intention is a phase III RCT of adding novel agents to the chosen regimen from InterAACT.

Recruitment rate was the main concern during design stage; anticipated enrolment is only 30 pts/year, despite international collaboration. The primary outcome measure is response rate, estimated as 40% with cisplatin + 5-FU. A clinically relevant improvement by 10–50% with carboplatin + paclitaxel required 388 patients/arm using a standard sample size calculation with 2-sided 5% significance level and 80% power; and an infeasible 25 years’ accrual. Researchers instead chose a Simon, Wittes and Ellenberg randomised selection trial design [4], requiring 40 patients/arm, for the same target difference and power. Completing accrual should take approximately 3 years. There is, however, limited protection of the Type 1 error in this design.

If the trials regimens have very similar response rates, the procedure will pick one by chance. The regimen with fewest severe toxicities will be accepted if the observed response rate is the same. Better quality-of-life (EORTC QLQ-C30, EQ-5D-5L) will decide if toxicity is also equal. If no winner is apparent after assessing activity, toxicity and QoL, a strong recommendation for which regimen to use in future phase III trials of combination therapy cannot be made.

### 2.2 Accept a greater type I error (salivary gland cancer)

The type I error is the probability of wrongly rejecting a null hypothesis (H_0_); erroneously concluding the research treatment is efficacious, active or interesting. This is traditionally 5% i.e. 1/20. The type II error is the probability of erroneously accepting the null hypothesis; missing an interesting treatment. A higher risk of type 2 errors is usually accepted, often 1/10 or 1/5, translating to 90% or 80% power, respectively.

One might decrease the required sample size by accepting a type I error more like a typical type II error. This approach is used in EORTC-1206-HNCG of salivary gland carcinomas (SGC), a heterogeneous group of rare tumours. SGC histologies constitute <5% of head and neck cancers. Patients treated with chemotherapy (CT) have low response rates and poor outcomes. There is compelling evidence from case series for sensitivity to androgen deprivation therapy (ADT) in androgen receptor-(AR)-expressing SGCs [5], [6], [7], [8]. Therefore, this trial compares ADT to CT in treatment-naive recurrent and/or metastatic SGC, restricted to salivary duct cancer and adenocarcinoma, two histologies where AR-expression is more common.

The primary outcome measure is progression-free survival (PFS). Patients must be treatment-naïve to evaluate PFS in the main study, but an exploratory substudy evaluates ADT in previously-treated patients.

The challenge was designing a trial with an acceptable compromise between (i) level of scientific evidence and (ii) feasibility in terms of trial size and duration.

Obtaining robust estimates of PFS for CT-treated patients was difficult: published studies were small and heterogeneous for histology, AR-expression and chemotherapy. The absence of good reference data strengthens the case for randomisation. Single arm and non-comparative randomised designs were dismissed before selecting a comparative randomised design. Discussion focused on the consequences of relaxing type I and II errors. As method of evaluating PFS, a time-to-event (PFS curve comparison) approach was selected over a binary (progression free rate at one time point relative to accrual) to avoid arbitrary time point selection. This choice contributed to reduction of sample size.

Pragmatic values for type I and II errors were selected: 80% power and 10% one-sided significance. The target effect size of hazard ratio (HR) 0.56 was chosen subsequently, based on 16 patients treated with ADT; it is equivalent to increasing 6-months PFS from 60% to 75%. The design requires 76 randomised patients over 2 years which was thought achievable after surveying interested institutions. Fifty-five PFS events are expected 1 year after accrual completes. The primary analysis is frequentist. A sensitivity analysis based on Bayesian methodology will assess the robustness of the conclusions for various prior distributions of the treatment effect hazard ratio.

### 2.3 Abandon a trial early for lack-of-benefit (uterine leiomyosarcoma)

There is an opportunity cost in continuing to assess a treatment that is unlikely to change practice, a cost felt more keenly in rare cancers. One may therefore consider interim futility analyses, which make assumptions about future data. This approach is used in uterine leiomyosarcoma (uLMS), a tumour of uterine muscle, prone to metastasising. Incidence is estimated at 0.4–0.64/100,000 women/year [9], [10]; 60% present with early stage disease. Uterine-confined, high-grade uLMS has a post-operative recurrence rate of 50–70% at 2–3 years [11], [12]. Observation after complete resection of uterus-limited disease is considered standard. Chemotherapy regimens reported to achieve good objective responses in metastatic disease include doxorubicin, doxorubicin and ifosfamide, gemcitabine, and fixed-dose rate gemcitabine and docetaxel.

Most published uLMS studies have been non-randomised. No adjuvant therapy has demonstrated improved survival. A single arm study of gemcitabine + docetaxel doublet followed by doxorubicin [11] reported 78% 2-year PFS and 57% 3-year PFS. This regimen was chosen for phase III comparison to observation until recurrence for women with high grade, uterus-limited, completely resected, LMS.

Assessing treatment arms from quite different modalities increases the challenge to recruitment. International participation was sought to maximise accrual. Recurrence-free survival (RFS) was initially chosen as the primary outcome measure, but there were concerns about potential bias in assessments in the absence of blinding, and blinding would not be feasible in a study where the control arm is observation. Therefore, survival was chosen as the primary outcome measure.

A high probability of early stopping for lack-of-benefit is particularly desirable given the disease rarity and contrast between trial arms. More outcome events (and thus statistical power) are available for RFS than survival at any time. Therefore, the probability of early termination under H_0_ is increased by replacing survival with RFS as the interim outcome measure for lack-of-benefit, with little or no power loss. This assumes that lack-of-benefit in RFS will translate to lack-of-benefit in survival, a reasonable assumption.

The interim futility boundary was set as HR ⩾0.90 in RFS to provide ⩾65% probability of early termination under H_0_. The loss in power for using RFS for the interim analysis depends on the correlation between the interim RFS and final survival test statistics. This correlation is not known in advance, but, with reasonable assumptions, the loss in power is estimated to be between 0.75% and 1.9%. This loss can be off-set by a small increase in observed number of deaths for the final analysis.

### 2.4 Test only research treatments with early discontinuation for lack-of-activity in absence of standard (metastatic uveal melanoma)

Despite aggressive local management of primary uveal melanoma (UM) with radiotherapy or surgical enucleation, metastases develop within 15 years in 50% patients [13], [14], [15]. Treatment has been improved for advanced cutaneous melanoma [16], [17], [18], a common condition, but no effective therapy has been approved for patients with the far rarer metastatic UM. This is highly-resistant to systemic therapy and prognosis is poor, with median PFS 2–4 months.

Multiple molecularly-targeted treatment strategies have been identified for clinical evaluation. Efficiently-designed clinical trial and multicenter collaborative efforts are required to test them.

No standard-of-care with demonstrable clinical activity is recognised against which experimental treatments can be compared. A control arm of no-treatment, perhaps placebo-blinded, may be justifiable but poses challenges for patient and physician acceptance; control with local standard management poses implementation challenges. Dacarbazine (DTIC) and temozolomide were considered internationally feasible control regimens, but a single-arm study of temozolomide [19] and RCT including DTIC and temozolomide demonstrated insufficient activity [20], [21].

Therefore, researchers agreed to assess two experimental arms: trametinib-alone or trametinib + AKT inhibitor. This two-arm randomised phase II study in 80 patients has a comparative final analysis and non-comparative interim analyses.

The primary outcome measure is PFS, defined as time from randomisation to the earliest of objective disease progression (Response Evaluation Criteria in Solid Tumours (RECIST)) or death from any cause. Median PFS trametinib is estimated as 16 wk. With 80 patients and 76 progression events, the probability is 80% of detecting a treatment difference at a one-sided 5% significance level if the true HR = 0.56. This assumes accrual of ∼24 months at 4 pt/m internationally, follow-up of 12 months. Patients will be assessed 8-weekly.

An early stopping rule for activity focuses on objective response rate (ORR; complete or partial response using RECIST) after recruiting 40 patients. Accrual will be terminated to either arm if <2 patients achieve ORR. The 95% confidence interval’s upper bound for 1/20 ORR is 25%. Any arm passing the interim analysis continues accrual to the pre-planned total of 40 patients. If there is just one such arm, it will be analysed as a single-arm phase II study and the PFS distribution assessed. The interim evaluation is justified by the rarity of disease and potential for inactive treatments.

### 2.5 Balance scientific value and feasibility (high-grade undifferentiated uterine sarcoma)

Opportunities to evaluate maintenance therapy are unusual for rare cancers, but this is addressed in high-grade undifferentiated uterine sarcoma (HGUS). Here, a randomised double-blind approach is used to evaluate maintenance therapy with cabozantinib after disease stabilisation or objective response to doxorubicin-based chemotherapy (CT).

Uterine sarcomas account for ∼1% female genital tract malignancies and 3–7% uterine cancers [22]. HGUS, constituting 6% of uterine sarcomas, has limited evidence concerning management; therapeutic strategies typically follow practice for other soft tissue sarcomas (STS) [23].

Prognosis of advanced HGUS is poor: median PFS <4 months, median survival <12 months. New regimens are needed. Disease stabilisation of STS can be achieved with pazopanib so anti-angiogenic agents represent an option. Maintenance therapy with anti-angiogenic agents, e.g. cabozantinib, may prolong the chemotherapy-induced response.

The literature provides no reliable guide to the efficacy of standard chemotherapy. The original concept was to enrol HGUS patients in the first-line setting, then randomise chemotherapy-responders to maintenance therapy with either study drug or placebo. A randomisation was envisaged for patients who recurred after first-line chemotherapy. This grand study design would address three objectives: the objective response to first-line chemotherapy; the effectiveness of maintenance treatment with an experimental anti-angiogenic agent; and, response and survival in patients treated with the experimental agent at time of disease progression. Such a study would reduce time and costs compared to three separate studies. Pharmaceutical industry support was obtained only for the maintenance therapy question, notably cabozantinib.

Recruitment to the maintenance study would be facilitated by permitting any first-line chemotherapy prior to study enrolment but investigators wanted some heterogeneity in first-line chemotherapy. Therefore, eligible patients must have received doxorubicin-based first-line chemotherapy. National groups will use standard regimens (limited to doxorubicin ± ifosfamide); a retrospective propensity-based [24] non-randomised evaluation of the additional ifosfamide is planned.

The resulting trial includes only one randomisation: cabozantinib versus placebo as maintenance therapy. An improvement in PFS is of clinical benefit in such a poor prognosis setting with limited treatments for recurrence; a 30% improvement in 4-months PFS from 50% to 80% is targeted. Survival and toxicity are key secondary outcome measures. 76 patients will be recruited, to randomise 54 patients, for 85% power to detect with a one-sided 15% significance level.

Cross-over to cabozantinib at progression is permitted which may appeal to participants, but complicates interpretation of survival data. The trial will also yield valuable translational research to inform further research.

### 2.6 Incorporate Bayesian elements to quantify resulting level of information (small bowel adenocarcinoma)

The previous designs each use a frequentist approach to the primary analyses. Bayesian designs are increasingly used, particularly in phase I studies [25] and adaptive designs [26], and may increase efficiency in these contexts. The Bayesian approach also has attractive features in rare cancers. It allows the incorporation of external information, including subjective information, into the estimation of treatment effects [27], supplementing the restricted information from the study itself [28], [29]. The BALLAD study mixes frequentist and Bayesian inferential frameworks.

BALLAD is a study in stage I–III small bowel adenocarcinoma (SBA), representing <5% gastrointestinal cancers. There were ∼2000 new cases and 400 deaths from SBA in the USA in 2008 [30], [31] The annual incidence is ∼0.22–0.57/100,000 inhabitants in Western countries [32]. Prognosis can be favourable and in stages I–III, ∼75% of diagnoses, are potentially curable [30].

There are no RCTs of adjuvant treatment following initial surgery in stage I–III SBA, but the proven benefits of fluoropyrimidine-based adjuvant therapy, with or without oxaliplatin, in colorectal cancer (CRC) suggest this may be worthwhile. Therefore, BALLAD aims to answer two questions in resected stage I–III SBA. Where the clinician is uncertain, what is the value of adjuvant post-operative chemotherapy over observation? Where the clinician is convinced of the value of adjuvant treatment, what is the value of adding oxaliplatin to adjuvant post-operative fluoropyrimidine-based chemotherapy?

The study design balances the need to produce persuasive evidence with the need for a sample size constrained by patient numbers and time. Four key design choices reflect these constraints.

First, disease-free survival (DFS) was selected as the primary outcome measure, rather than survival. This reduces the sample size as DFS events occur earlier; evidence from the colorectal cancer setting supports surrogacy of DFS for survival.

Second, a conventional (frequentist) design would ideally have been selected, but the power and significance levels for traditional design resulted in an infeasible target of ∼1500 patients. A hybrid approach was therefore selected, using a standard randomised phase II design, and using Bayesian techniques to incorporate subjective clinician estimates of treatment effect based on external evidence. This hybrid approach requires some objective statistically-significant evidence of treatment benefit from the study data; the study is designed to have 80–90% power to detect HR = 0.75 for each question at the 20% 1-sided significance level. If the results are statistically significant at 20%, the data will be combined with clinician estimates of the treatment benefit, based on their interpretation of external evidence from the relevant literature to provide an overall combined estimate. These clinician estimates will be obtained in the first year of recruitment [33].

Third, to facilitate recruitment, flexibility is allowed in the choice of fluoropyrimidine. Patients allocated to chemotherapy in the first randomisation may also be randomised to receive oxaliplatin or not, boosting recruitment to the second randomisation and the overall efficiency of the design.

Finally, the study may be stopped early for futility when half the events have been observed. Recruitment will be in the range of 545–860 patients. The success of recruitment to each randomisation will be assessed independently.

### 2.7 Bayesian design with reverse philosophy (squamous carcinoma of the penis [InPACT])

A key motivation for Bayesian designs is focusing on estimation rather than hypothesis testing; trial data are used to reduce uncertainty about the size of the treatment effect and inform future clinical practice [29]. A ‘reverse philosophy’ means starting with the number of patients and events that could be feasibly recruited and accumulated within a timescale, then assessing whether that amount of data would have sufficient value to justify a trial. Such feasibility issues are more prominent in rare diseases.

The design evaluates the clinical utility of the trial by (i) demonstrating the information that a Bayesian analysis could provide for a range of possible observed trial results and prior distributions; and (ii) assessing the operating characteristics of the design i.e. the chance of erroneous conclusions from typical decision criteria under a range of underlying true scenarios using simulation.

The InPACT trial uses this approach for patients with inguinal lymph node metastases from squamous carcinoma of the penis i.e. locally advanced disease. The annual incidence in the UK is 1.5/100,000, with 558 new cases in 2011 and 106 deaths in 2012 [34]. There were 1570 new cases and 310 deaths in the USA in 2013 [35].

The trial has two independent randomisations, addressing key questions in the clinical pathway: First, the role of neoadjuvant therapy prior to standard surgery, by randomising to chemotherapy, chemoradiotherapy, or no neoadjuvant therapy. Second, the role of prophylactic pelvic lymph node dissection (PLND) in only higher-risk patients following their standard surgery with therapeutic inguinal lymph node dissection (ILND). The primary outcome measure is overall survival.

The trial will accrue for 5 years with 2 years follow-up. This should yield at least 400 patients, with 176 patients contributing to the neoadjuvant therapy question, including 132 to the sub-question of type of neoadjuvant therapy, and 240 to the PLND question. The predicted numbers of deaths for the analyses are 88/176, 84/132 and 181/240, respectively. The posterior probability distributions should provide sufficient certainty (86% on average across different questions and different priors) that the treatment is effective to inform clinical practice, if a modest treatment effect is observed (i.e. HR = 0.8). There is a high probability (81% on average) of selecting the right treatment, using Bayesian decision rules, if a treatment has a true modest, clinically-relevant effect (HR = 0.8); and a low probability (8% on average) of accepting a treatment when there is a true negative effect (HR = 1.25). There is a moderately high chance (39% on average) of incorrectly accepting the ‘experimental treatment’ for future use if there is no true effect (HR = 1); this is considered an acceptable trade-off.

The design uses four off-the-shelf prior distributions (non-informative, sceptic, extreme sceptic, enthusiast), incorporating the equivalent of 20 deaths to represent weak prior evidence or beliefs in the latter three cases. The trialists intend to perform a systematic review of the literature in order to create relevant evidence-based priors for use in the final analysis.

### 2.8 Multiple concurrent treatments, interim analysis based on merit

Most trials randomise patients between only two arms, but, in some circumstances, there are more agents, combinations or other approaches to treatment suitable for testing. How should researchers select which to take forward into their new two-arm trial? There are opportunity costs in pursuing just one treatment, particularly if results are not positive, and these are felt even more keenly in rare diseases.

An alternative to multiple parallel 2-arm trials is a single multi-arm trial where a series of research arms are assessed in parallel against a common control arm. The control arm patients efficiently contribute to each pairwise comparison and only one protocol is required.

This approach is extendable with intermediate stopping rules, like those in the uterine leiomyosarcoma study, which stop recruitment early to insufficiently active research arms. One implementation is the multi-arm multi-stage (MAMS) approach where recruitment is stopped early to research treatments that are insufficiently active based on pre-specified lack-of-sufficient-benefit analyses [36], [37] Recruitment continues based only on merit in the observed data; recruitment would completely stop early only if all research arms showed a lack-of-sufficient-benefit; or would continue to the control arm and any research arms that look sufficiently interesting. Researchers would need to be prepared to recruit to the maximum number of patients, although this is unlikely given the unfortunate situation that most new treatments work less well than hoped [38].

This approach is successfully implemented in common diseases [39], [40] and potential trials have been developed in rare cancers, notably osteosarcoma and ocular melanoma, where investigators saw it as an efficient approach. Access to the necessary drugs was problematic for an ocular melanoma proposal, but the European and American Osteosarcoma Study (EURAMOS) Strategy Group remains committed to a multi-arm multi-stage international comparison in the future when further early phase studies have been completed. The multi-arm approach has been supported in rare diseases outside of cancer e.g. erosive lichen planus of the vulva, a very rare dermatological condition, where a four-arm trial (three research arms) will commence recruitment in the UK in 2014. Although there are no examples of its implementation in rare cancers so far, the design is a weapon in our armamentarium.

### 2.9 Multi-arm selection without assumption (Ewing sarcoma)

Assuming the efficiency of running multi-arm trials, alternative ways of selecting which treatments should continue to be assessed may be investigated. These could incorporate randomised selection designs and probability-based interpretation, using Bayesian posterior probability distributions with a non-informative prior. This approach is used in Ewing sarcoma, a rare cancer mainly in children and young adults. Initial treatment leads to event-free survival (EFS) of ∼50% at 5 years [41]. Prognosis in both refractory disease after induction chemotherapy and after recurrence is very poor. Several chemotherapy regimens are used at recurrence, but there is no randomised evidence driving treatment decisions; only limited data from small single-arm series, making comparison of regimens very unreliable. The four re-induction regimens most widely used are: topotecan + cyclophosphamide (TC), irinotecan + temozolomide (IT), gemcitabine + docetaxel (GD) and high-dose ifosfamide alone (IFOS).

The rEECur trial adopts an adaptive multi-arm design with forced selection at a series of pre-specified interim analyses. The initial Phase II stage, with response as the primary outcome measure, randomises 50 patients to each of four arms: TC, IT, GD and IFOS. Recruitment to one arm will be stopped at that point and to a second arm after a further 25 patients/arm. The remaining two arms will continue to a Phase III evaluation with EFS as the primary outcome measure.

The probabilities of ‘dropping’ the best arm at each stage have been calculated under various scenarios. For example: if the true response rate is 50% for the best arm and 40% for the other arms, there are 3% and 5% chances of erroneously dropping the best arm after 50 and 75 patients/arm, respectively; the cumulative probability of 8%. Response is the primary intermediate outcome measure but other factors (toxicity and convenience) will be considered in decision-making. The Independent Data Monitoring Committee (IDMC) will make this decision.

The Phase III element adopts a probability-based approach to interpretation. The sample size calculation for a conventional design, with 2-sided alpha 5% and 80% power, requires 660 patients for an absolute difference of 10% in EFS. All are accepted regimens so there is no clinical need to be 97.5% certain that one regimen is better than the other. If there is, say, an 80% chance that EFS is better with one regimen (equivalent to 2-sided *p* = 0.4) with little difference in toxicity, the clinical community would accept this as the standard-of-care for future trials. The decision as to which regimen is the better after the final analysis will also take account of all relevant information.

The researchers see rEECur as the beginning of an ongoing programmatic collaboration, with new regimens introduced for assessment, as new arms or as factorial comparisons.

## 3 Discussion

The IRCI trialists have contributed to a joint discussion of the key parameters and main concerns in designing and executing clinical trials in rare cancer populations. Section 2 discussed some of the thinking behind the diverse approaches that were selected. We summarise the joint themes coming from the discussion.

The diseased population available for sampling limits the application of the laws of probability as applied in biostatistics but the laws do not change because of rarity. One main role of statistics in the set-up and interpretation of clinical trials is to delineate the uncertainty around the results. The previous examples illustrate many approaches to performing credible clinical trials.

What makes a credible trial in rare cancers?

*Comparative data:* The lack of robust historical data greatly affects the starting point of trial design. Absence of an evidence-based standard-of-care commonly leads to heterogeneity in practice. Design assumptions often use small series or extrapolation from other settings. There may be little agreement on standard parameters such as prognostic factors and expected outcomes. Randomisation becomes a must-have in such situation, allowing causation and the establishment of some levels of evidence. Indeed, randomisation is usually required for inclusion in the IRCI network.

*Correct quantification of risks:* A second element is correct delineation of the resulting error margins. Whether in a frequentist or Bayesian interpretation, trialists need to agree upfront on acceptable false positive (type 1 error) and false negative (type 2 error) rates. A frequently used ploy in rare cancer trials is to soften error rates, thus restricting samples sizes at the expense of higher risks of false conclusions.

*The result in the external world:* The third and final element for a credible trial is the hardest one, and is in the eye of the beholder. Regulators, clinicians, patients, pharmaceutical companies and payers all have their own angle of interpretation, as in common diseases. The differences in position between these stakeholders may be particularly apparent with increased uncertainty. An RCT ideally leads to the same interpretation by all stakeholders, matching the intent of the trialists, but this may be a utopian view. However, international collaboration, and upfront agreement on the trial settings by a large body of partners, upfront involvement of regulators and patient organisations can only help.

To further specify, as for any trial, the objective is essential. The conclusion sought may be definitive or practice-changing, a phase III-type question, or it might be feasibility, activity or treatment selection, a phase II-type question. The former faces considerable hurdles in the rare disease setting. For more common cancers, the ‘rules’ are – at least implicitly – fairly well agreed within the community, and, ultimately, the regulator.

We advocate that if a trial is meant to be definitive or practice-changing (i.e. no follow-on trial of the same question is expected), it should be labelled as a Phase III trial, despite pragmatic compromises on sample size, error margins and other design elements. If all efforts – including wide international collaboration—have been made to enable the largest enrolment, the community will need to interpret the results as potentially practice defining. It is essential to seek broad consensus within the expert community that the trial, if positive, would change practice, and to seek concurrence of regulators and other stakeholders. In some other situations, a randomised Phase II trial can give the community the necessary confidence to run the true next Phase III trial as a follow-up.

In rare cancer trials, we want to warn against some misconceptions. The first is a blanket appeal for ‘statistical efficiency’, suggesting that more can be achieved by trying harder. This is a misnomer, because any clinical trial carried out without rigour and without a proper design would be unethical. The call for efficiency is a placeholder for issues that are of importance in any clinical trial, but tend to be exacerbated by expectation of low accrual and knowledge that few trials can be undertaken in the population. There are multiple ways to balance the scarcity of patients and the objective of the trial. The variety of approaches at the IRCI workshop was striking, and we have illustrated these, above.

The choice of outcome measures will critically affect trial design. Intermediate or potentially surrogate end-points allow smaller trials but an inappropriately early outcome measure must not be selected just to reduce the trial’s size. Multiple or staged outcome measures may be considered. Matters of multiple testing should be explicitly discussed. Recruitment may be stopped early to arms that are underperforming; the opportunity costs in chasing false hopes are amplified in rare cancers. Early stopping rules, using lack-of-activity or futility-based measures, might be better accepted by trial teams in the context of multi-arm trials where the hard-won research activity is majorly altered rather than stopped completely.

Care must be taken in relaxing the type I and type II errors, especially if the intent is to change practice. It seems rational to allow a higher type I error where few options are open in the foreseeable future and a higher type II error if many candidate treatments exist. Again, this risk is to be embraced by the relevant clinical community and the regulators to ensure that efforts are worthwhile.

A second, more opaque danger in rare cancer trials is to try to ask too many questions in a single trial. In the absence of good prior scientific data, it is a dangerous choice to try for too much. In common cancers, most definitive studies follow a traditional design with respect to patient population, course of disease and line of therapy. Many questions are typically open for any rare disease. The prospect of a trial in a rare disease can make researchers try to tackle all relevant questions simultaneously. This may lead to loss in focus, inability to define the primary question, delayed development and funding problems. The trials discussed above are all more or less open to such criticism, reflecting the need for clinical trials and the risk of overburdening the design.

We cannot discuss here the practical challenges in conducting clinical trials across multiple countries but note that international collaboration is a considerable challenge, even when there is desire from the researchers. Navigating the regulatory requirements from various authorities can be daunting but necessary for success. We also cannot discuss translational research, but all trials must collect and process samples in a coordinated way in order to understand the diseases and investigate biological data with consistently collected prospective data.

## 4 Conclusion

The challenges and risks in designing and conducting a trial to provide sufficient evidence remain enormous in rare diseases, despite the constraints of sample size and insufficient background information. Trial designs can be undertaken using a wide array of possibilities, as illustrated. There is no ‘one size fits all’ solution. We have set out some examples of approaches accepted by IRCI so far so that future researchers might consider the relative positives and negatives.

In order to make progress in the treatment of rare diseases, decisions to change practice will have to be based on less direct evidence from clinical trials than in more common diseases. Approaches to aid decision making by augmenting direct randomised trial evidence in a structured manner with external sources should be explored.

## Disclosure

This manuscript is based on a meeting of IRCI methodologists held in Amsterdam in Sep-2013 ahead of the European Cancer Conference.

## Support

Matthew Sydes time was funded by the 10.13039/501100000265Medical Research Council through University College London (UCL).

## Conflict of interest statement

Clare Peckitt received an honorarium from Sanofi. All other authors disclose no conflicts of interest.

## Footnotes

3 **Associations: InterAACT/IRCI 003;** Sheela Rao (Chief Investigator), Clare Peckitt (Trial Statistician), Francesco Sclafani, Al Benson, Dirk Arnold, Cathy Eng, Rob Glynne-Jones. **ADT in advanced SGCs/IRCI 007;** Lisa Licitra (Chief Investigator), Catherine Fortpied (Trial Statistician), Alan Ho, Kevin Harrington.**GOG-0277/IRCI 001;** Martee Hensley (Chief Investigator), Virginia Filiaci (Trial Statistician), Helen Hatcher, Jean-Yves Blay. **MEKi +/- AKTi in UM/IRCI 005;** Richard Carvajal (Chief Investigator); Kathy Panageas (Trial Statistician), Ernie Marshall, Poulam M. Patel, Serge Leyvraz. **HGUS/IRCI 006;** Isabelle Ray-Coquard (Chief Investigator), Corneel Coens (Trial Statistician), Helena Earl. **BALLAD/IRCI 002;** Richard Wilson (Chief Investigator), James Paul (Trial Statistician), Arnaud Roth, Rob McWilliams. **InPACT/IRCI 004;** Steve Nicholson (Chief Investigator), Lucinda Billingham & Emma Hall (Trial Statisticians), Curtis Pettaway. **rEECur;** Martin McCabe (Chief Investigator), Keith Wheatley (Trial Statistician), Jeremy Whelan.
